# Supplementary material for: MSH2 stimulates interfering and inhibits non-interfering crossovers in response to genetic polymorphism
Source: Nat Commun. 2023 Oct 23;14:6716. doi: 10.1038/s41467-023-42511-z (PMC10593791; doi:10.1038/s41467-023-42511-z)
Supplement: Supplementary file 1 — Supplementary Information [file 41467_2023_42511_MOESM1_ESM.pdf]

MSH2 stimulates interfering and inhibits non-interfering crossovers in response to genetic polymorphism

Julia Dluzewska, Wojciech Dziegielewski, Maja Szymanska-Lejman, Monika Gazecka, Ian R. Henderson, James Higgins and Piotr A. Ziolkowski

Supplementary Information

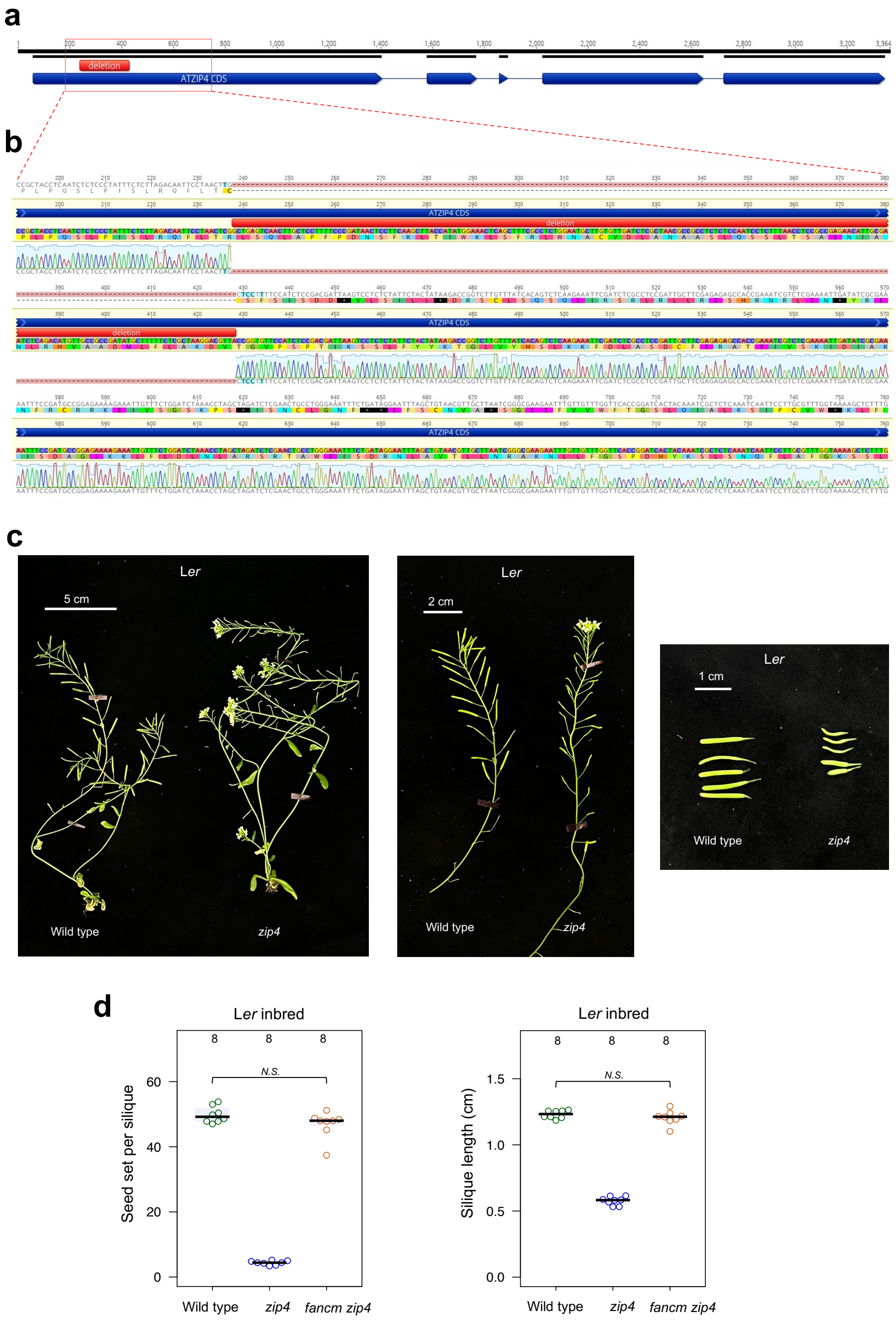

Supplementary Fig. 1. *ZIP4* CRISPR/Cas9-mediated mutagenesis in L*er* accession. **a**, ZIP4 coding sequence with exons represented in dark blue arrows. Mutagenesis by CRISPR/Cas9 resulted in 191 bp deletion within the first exon (annotated as a red square) and introduction of 5 SNPs within the Cas9 cutting sites. **b**, Close-up view of the L*er zip4* sequencing aligned to the wild-type reference shows that the 191 bp deletion results in a frameshift and multiple STOP codons, represented in black. **c**, The L*er zip4* mutant obtained by CRISPR/Cas9 shows dramatically reduced fertility. Whole plants (left) are shown next to primary shoots (center) and exemplary siliques (right). **d**, Fertility assays in L*er zip4* and *fancm zip4* as assessed via seed set and silique length. The center line of a boxplot indicates the mean; the upper and lower bounds indicate the 75th and 25th percentiles, respectively. Each dot represents a measurement from five siliques of one plant. The numbers of plants are indicated above the boxplots. Significance was assessed by Welch's *t*-test. Source data are provided as a Source Data file.

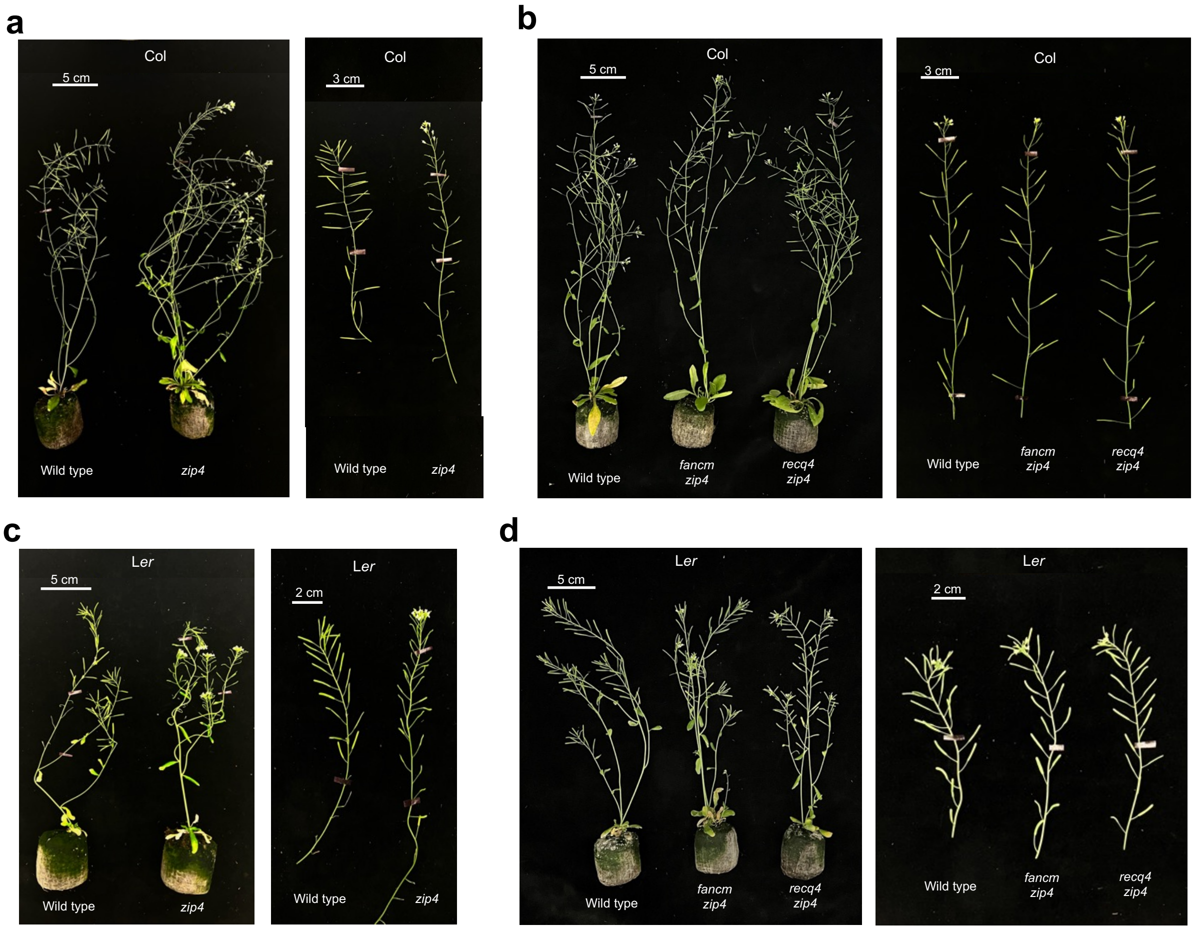

Supplementary Fig. 2. Fertility, which is lost in Col and L*er* *zip4* mutants, is restored in *fancm* or *recq4* (*recq4a recq4b*) mutant backgrounds. **a**, comparison of wild type and *zip4* plants (left) and shoots (right) in Col background. **b**, as in **a**, but for the *fancm zip4* and *recq4 zip4* mutants. **c**, as in **a**, but in the L*er* background. **d**, as in **a**, but for the *fancm zip4* and *recq4 zip4* mutants in L*er* background.

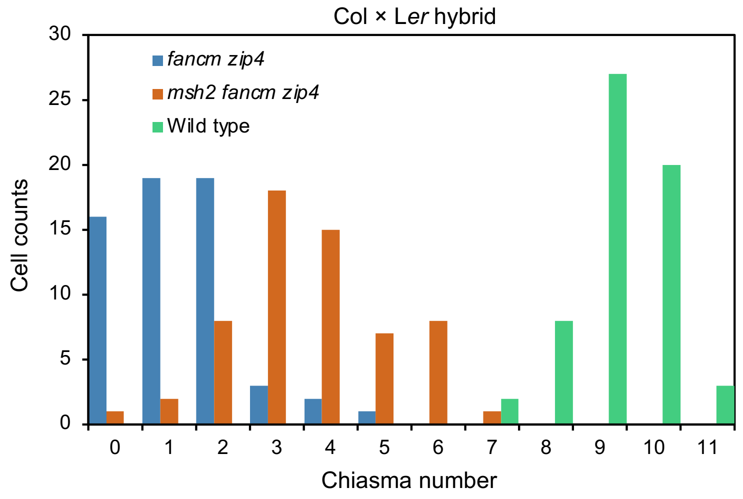

Supplementary Fig. 3. Quantification of chiasma count data from Col × L*er* male meiocytes in wild type, *fancm zip4* and *msh2 fancm zip4*. Source data are provided as a Source Data file.

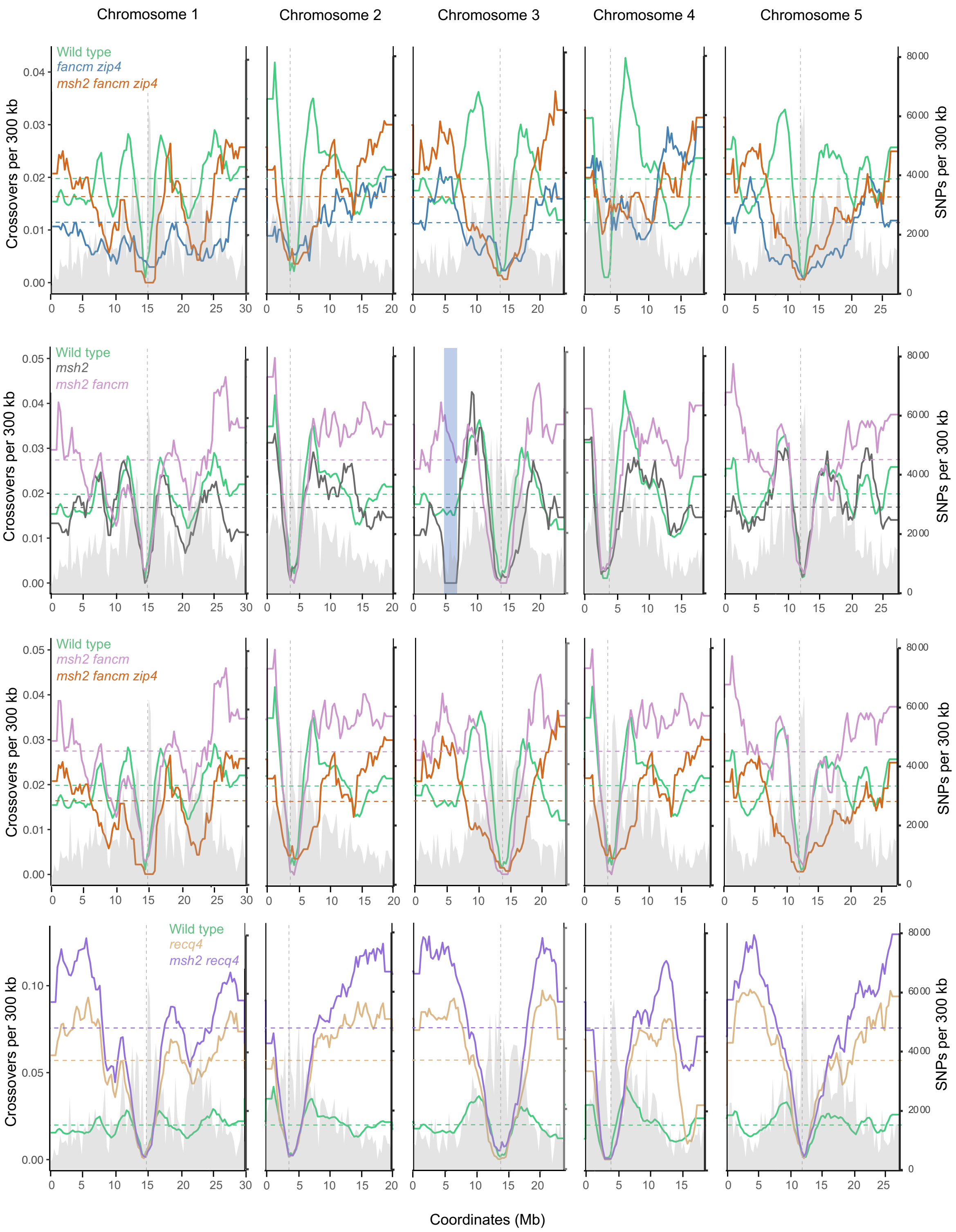

Supplementary Fig. 4. Crossovers per 300 kb per F_2 plotted along the Arabidopsis chromosomes. Mean values are shown by horizontal dashed lines. SNPs per 300 kb are plotted and shaded in gray. The position of centromeres is indicated as vertical dashed line. The introgression of Col in *msh2* L*er* due to *msh2* backcrossing results in failure to detect crossovers, which is indicated as a blue rectangle. Source data are provided as a Source Data file.

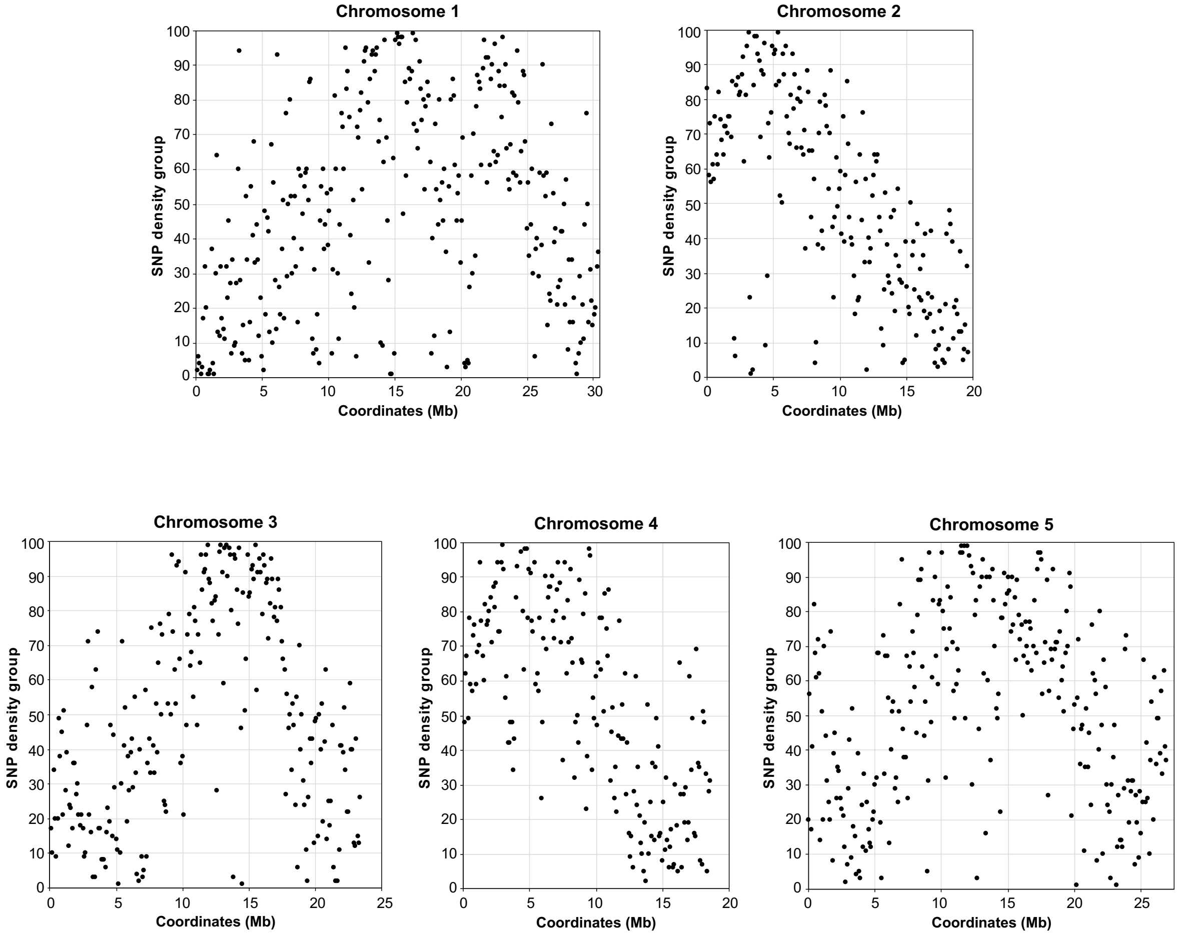

Supplementary Fig. 5. Chromosomal distribution of 100 kb windows for each SNP density group used in Figure 3. Each dot corresponds to one window, each group (Y-axis) consists of 12 windows. Source data are provided as a Source Data file.

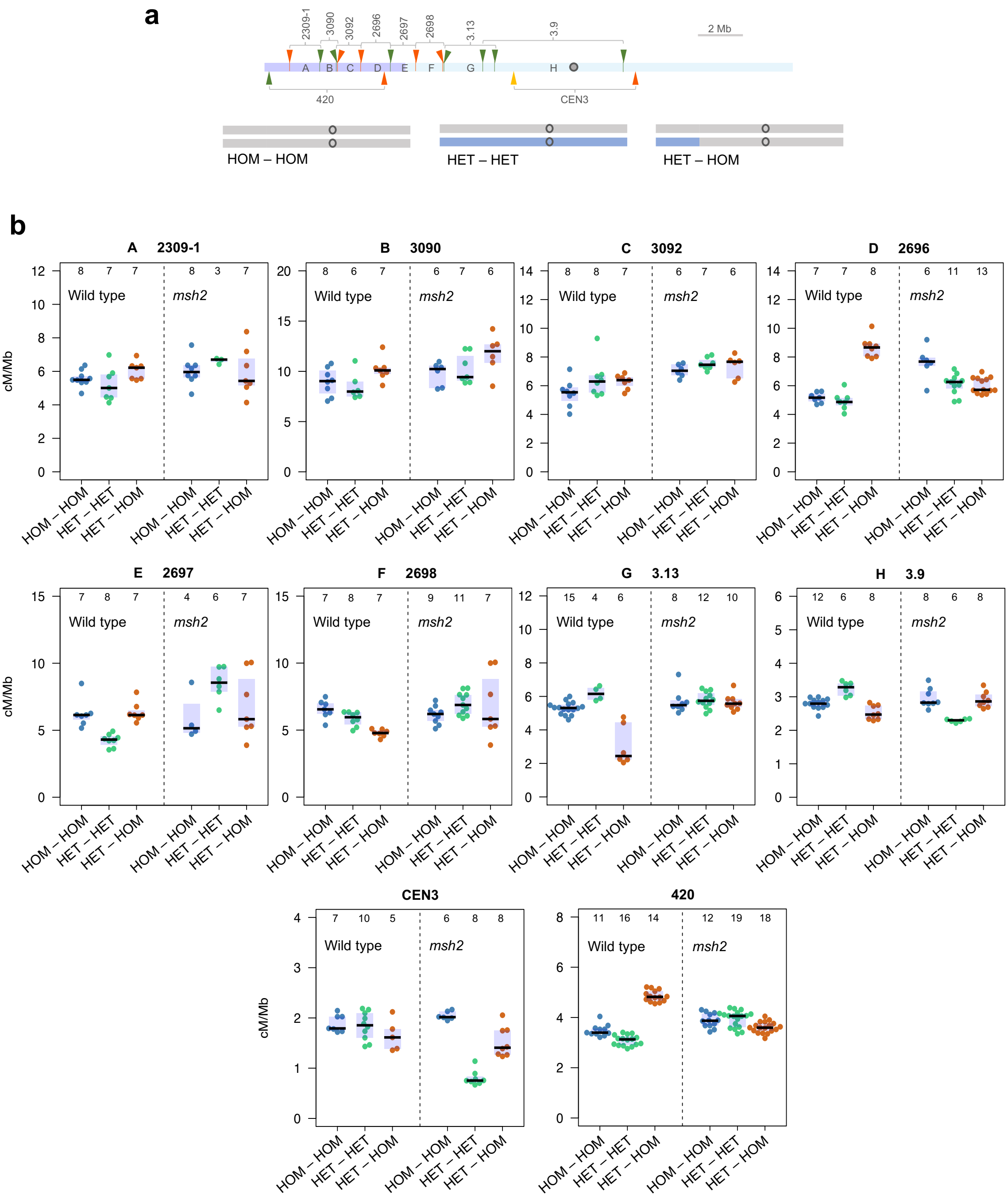

Supplementary Fig. 6. Crossover distribution across the chromosome arm in response to polymorphism. The plots show the same data as in Figure 5, but separately for each FTL. **a**, Location of eight FTL intervals across the Arabidopsis chr. 3 shown in addition to *420* and *CEN3* intervals. Fluorescent reporters are indicated by tick marks and arrowheads with colors corresponding to eGFP (green), dsRed (red) and eYFP (yellow). The violet and light blue shading represents 'HET' and 'HOM' regions in HET-HOM line, respectively. Genetically defined centromere[1] is indicated as gray circle. Below, ideograms of chr. 3 showing heterozygosity pattern in lines used in **b**. Gray and blue correspond to Col and Ct genotype, respectively. **b**, Crossover frequency (cM/Mb) in the HOM-HOM, HET-HET and HET-HOM genotypes shown separately for ten FTLs, in either wild type or *msh2*. The center line of a boxplot indicates the mean; the upper and lower bounds indicate the 75th and 25th percentiles, respectively. Each dot represents a measurement from one individual. The numbers of individuals are indicated above the boxplots. Source data are provided as a Source Data file.

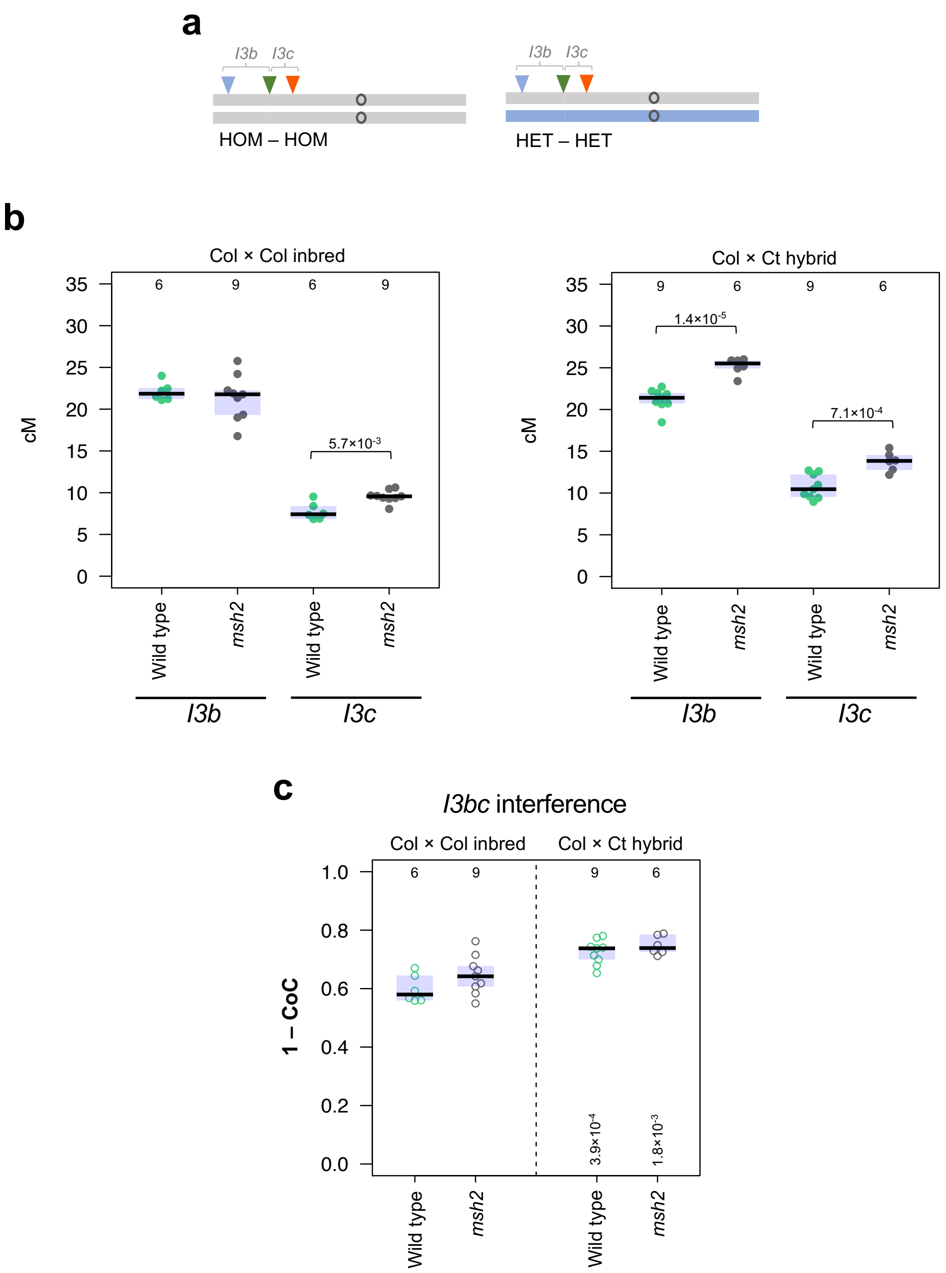

Supplementary Fig. 7. Crossover interference is not changed in *msh2* inbreds and hybrids. **a**, Ideograms of chromosome 3 showing the location of *I3b* and *I3c* intervals in Col/Col inbred and Col/Ct hybrid plants used in the experiment. Gray corresponds to Col while blue corresponds to Ct genotype. Location of fluorescent reporters defining *I3b* and *I3c* interval is indicated by blue, green and red triangles. **b**, *I3b* and *I3c* crossover rate as measured in Col/Col inbred and Col/Ct hybrid plants as measured in wild type and *msh2* plants. Welch's t-test was used to calculate statistical significance. **c**, Crossover interference (1 – CoC) as measured over *I3bc* intervals for Col/Col inbred and Col/Ct hybrid plants in wild type and *msh2* plants. Two-sided *P* values indicate the statistical difference in crossover interference in hybrids versus inbreds (Welch's t-test). Each dot on **b** and **c** represents a measurement from a pool of 5-8 individuals. The numbers of pools are indicated above the boxplots. Source data are provided as a Source Data file.

Supplementary Table 1. List of oligonucleotides.

ID	Primer sequence	Comments
**JZ-001**	TTAAACTCACTTGGCAATCGC	*msh2-1* SALK mutant in Col, use together with LBb1.3
**JZ-002**	TAAGCTTTGCTGATTTGGCTG	
**JZ-14**	AACCACTGCTCTACGTCAGC	*msh2* CRISPR/Cas9 deletion mutants genotyping
**JZ-15**	TCGCTTCAGATGCTGTCTAA	
**zip4-F**	TTGCTACCTTGGGCTCTCTC	*zip4-2* SALK mutant in Col, use together with LBb1.3
**zip4-R**	ATTCTGTTCTCGCTTTCCAG	
**zip4_CRISPR_F**	TCTCCCGCCAACAATCGAAA	*zip4-3* CRISPR/Cas9 deletion mutant genotyping in L*er*
**zip4_CRISPR_R**	CTGCAATCGTCGTCACCTCT	
**fancm-dCAPS-F**	ACAATATATGTTTCGTGCAGGTAAGA CATTGGAAG	*fancm-1* mutant in Col, dCAPS primers: digestion with MboII enzyme
**fancm-dCAPS-R**	CACCAATAGATGTTGCGACAAT	
**fancm-Ler-L**	GCTTGGTGATCGACGAGGCACATCAA G	*fancm-10* mutant in L*er*, dCAPS primers: digestion with HindIII enzyme
**fancm-Ler-R**	ATTAGTTGGTCACCTTGATCAT	
**recq4a-F**	ATCAGAGCCACTCATTGTTG	*recq4a-4* mutant in Col, multiplex PCR
**recq4a-wt-R**	GTCCTGATCGTGTTGGACAG	
**recq4a-mut-R**	ATATTGACCATCATACTCATTGC	
**recq4b-wt-F**	TCAGAAAGTTGCTCTGCGTC	*recq4b-2* wild type allele in Col
**recq4b-wt-R**	ACCAAGACCCTGCATATTGC	
**recq4b-mut-F**	ACTAGAGATACTTCAGGAGCTGAGC	*recq4b-2* mutant allele in Col
**recq4b-mut-R**	GCTTTCTTCCCTTCCTTTCTC	
**recq4a_Ler_F**	GACCAAGGCAGATATGCCTGTGATAC CTG	*recq4a* mutant in L*er*, dCAPS primers: digestion with ScrFI enzyme
**recq4a_Ler_R**	GTCCAGGGAAATTCACGACTGGTC	
**LBb1.3**	ATTTTGCCGATTTCGGAAC	Universal primer for SALK mutants
**HEI10_C2_F**	TAATACGACTCACTATAGGG	Genotyping of *HEI10-OE* construct presence in C2 line
**HEI10_C2_R**	GCCAGCAAGACAGAACAGTTC	
**ZIP4-gRNA1**	AAGTTGACTCAGCCGAGTT	gRNAs binding within 1st exon of *ZIP4*
**ZIP4-gRNA2**	AATCGTCGGAGATGGAACAC	

Supplementary Table 2. Characteristics of a CTL panel (*2309-1*, *3090*, *3092*, *2696*, *2697*, *2698*, *3.13*, *3.9*) overlapping norther arm and centromeric region of chromosome 3. CTLs were created by Wu et al. (2015).

**CTL name**	**Size (Mb)**	**Single-color reporter line**		**Coordinates (bp)**	
		**Left**	**Right**	**Left**	**Right**
2309-1	1.36	CR92	CG50	1 171 464	2 528 742
3090	0.77	CG50	CR352	2 528 742	3 300 254
3092	1.05	CG21	CR73	3 280 460	4 330 342
2696	1.27	CR73	CG24	4 330 342	5 603 768
2697	1.16	CG24	CR1045	5 603 768	6 761 383
2698	1.26	CR1045	CG43	6 761 383	8 017 809
3.13	2.25	CR729	CG906	8 072 294	10 318 203
3.9	6.24	CG17	CR55	9 741 508	15 980 483

Supplementary Table 3. Summary of the GBS libraries and crossovers identified. Data for *fancm zip4*, *msh2 fancm*, *msh2 fancm zip4* and *msh2 recq4*, where generated in this work, while data for wild type, *msh2* and *recq4* mutants were adopted from previously published reports (references indicated next to genotype name).

Genotype	Total crossover number	Total reads	No. of samples	Avg. reads per library	Avg. coverage per library
fancm zip4	1,061	109,121,099	232	470,349	1.23
msh2 fancm	1,976	117,537,405	201	584,763	0.93
msh2 fancm zip4	1,324	69,456,952	184	377,483	1.81
msh2 recq4	8,573	196,367,255	283	693,877	1.40
Wild type[2]	14,998	518,905,436	1859	279,131	0.73
msh2[3]	1,278	100,524,854	188	534,707	1.39
recq4[4]	4,389	115,931,182	192	603,808	1.58

Supplementary references

1. Copenhaver, G. P. *et al.* Genetic definition and sequence analysis of Arabidopsis centromeres. *Science* **286**, 2468–74 (1999).
2. Rowan, B. A. *et al.* An Ultra High-Density Arabidopsis thaliana Crossover. *Genetics* **213**, 771–787 (2019).
3. Blackwell, A. R. *et al.* MSH2 shapes the meiotic crossover landscape in relation to interhomolog polymorphism in Arabidopsis. *EMBO J.* **39**, e104858 (2020).
4. Serra, H. *et al.* Massive crossover elevation via combination of HEI10 and recq4a recq4b during Arabidopsis meiosis. *Proc Natl Acad Sci U S A* **115**, 2437–2442 (2018).
