# Supplementary material for: MSH2 stimulates interfering and inhibits non-interfering crossovers in response to genetic polymorphism
Source: Nat Commun. 2023 Oct 23;14:6716. doi: 10.1038/s41467-023-42511-z (PMC10593791; doi:10.1038/s41467-023-42511-z)
Supplement: Supplementary file 3 — Reporting Summary [file 41467_2023_42511_MOESM3_ESM.pdf]

nature portfolio

Corresponding author(s): Piotr A. Ziolkowski

Last updated by author(s): Oct 12, 2023

Reporting Summary

Nature Portfolio wishes to improve the reproducibility of the work that we publish. This form provides structure for consistency and transparency in reporting. For further information on Nature Portfolio policies, see our Editorial Policies and the Editorial Policy Checklist.

Statistics

For all statistical analyses, confirm that the following items are present in the figure legend, table legend, main text, or Methods section.

n/a	Confirmed	
☐	☒	The exact sample size (*n*) for each experimental group/condition, given as a discrete number and unit of measurement
☐	☒	A statement on whether measurements were taken from distinct samples or whether the same sample was measured repeatedly
☐	☒	The statistical test(s) used AND whether they are one- or two-sided<br>*Only common tests should be described solely by name; describe more complex techniques in the Methods section.*
☐	☒	A description of all covariates tested
☐	☒	A description of any assumptions or corrections, such as tests of normality and adjustment for multiple comparisons
☐	☒	A full description of the statistical parameters including central tendency (e.g. means) or other basic estimates (e.g. regression coefficient) AND variation (e.g. standard deviation) or associated estimates of uncertainty (e.g. confidence intervals)
☐	☒	For null hypothesis testing, the test statistic (e.g. *F*, *t*, *r*) with confidence intervals, effect sizes, degrees of freedom and *P* value noted<br>*Give P values as exact values whenever suitable.*
☒	☐	For Bayesian analysis, information on the choice of priors and Markov chain Monte Carlo settings
☒	☐	For hierarchical and complex designs, identification of the appropriate level for tests and full reporting of outcomes
☒	☐	Estimates of effect sizes (e.g. Cohen's *d*, Pearson's *r*), indicating how they were calculated

Our web collection on statistics for biologists contains articles on many of the points above.

Software and code

Policy information about availability of computer code

Data collection	No software was used to collect the data. Computer code used are primarily custom R scripts (version 4.2) and CellProfiler (version 4.07)
Data analysis	Identification of crossover breakpoints.<br>To identify Single-nucleotide polymorphisms (SNPs) within the tested populations, demultiplexed paired-end forward and reverse reads have been pooled and aligned to Col-0 genome reference sequence with use of BowTie2 (Langmea et al., 2013). Resulting BAM files have been sorted and indexed with use of SAMtools v1.2 (Li and Durbin, 2009). SNPs were called using SAMtools and BCFtools. Subsequently, individual sequencing libraries have been aligned to Col-0 genome reference sequence with default parameters in BowTie2 and compared to previously generated SNP list with SAMtools and BCFtools. Later, the resulting tables of SNPs have been filtered to keep only SNPs with high mapping quality (>100) and high coverage (>2.5×) in R. Individual libraries with less than 100,000 reads were discarded from the analysis. To call crossovers TIGER pipeline has been used on filtered files (Rowan et al., 2015). To investigate CO distribution, crossover frequencies have been binned into scaled windows and summed across chromosome arms.<br><br>Analysis of the relationship between crossover recombination and SNP density.<br>Arabidopsis genome was divided into 100 kb non-overlapping windows and for each of them SNP density was determined based on published Col/Ler polymorphism data (Zapata et al., 2016). The crossover frequency per each window was normalized to the number of analyzed individuals. This resulted in 1191 windows, which were sorted according to the SNP density and grouped into 99 groups, so that each group consisted of 12 windows with a similar polymorphism level. This analysis was performed in Microsoft Excel.

For manuscripts utilizing custom algorithms or software that are central to the research but not yet described in published literature, software must be made available to editors and reviewers. We strongly encourage code deposition in a community repository (e.g. GitHub). See the Nature Portfolio guidelines for submitting code & software for further information.

Data

Policy information about availability of data

All manuscripts must include a data availability statement. This statement should provide the following information, where applicable:

- Accession codes, unique identifiers, or web links for publicly available datasets
- A description of any restrictions on data availability
- For clinical datasets or third party data, please ensure that the statement adheres to our policy

All data generated for this study are included in the published version of the article, or its supplementary data files. The GBS sequence data generated in this study (for msh2 fancm, msh2 fancm zip4, msh2 recq4ab and fancm zip4) have been deposited in the NCBI Sequence Read Archive (SRA) under the BioProject accession codes PRJNA952840 [https://www.ncbi.nlm.nih.gov/bioproject?LinkName=sra_bioproject&from_uid=27261978]. Raw GBS data for Col x Ler F2 wild type population have been downloaded from ArrayExpress E-MTAB-8165 [https://www.ebi.ac.uk/biostudies/arrayexpress/studies/E-MTAB-8165?query=E-MTAB-8165] (Rowan et al., 2019). Raw GBS data for Col x Ler F2 recq4ab mutant have been downloaded from ArrayExpress E-MTAB-5949 [https://www.ebi.ac.uk/biostudies/arrayexpress/studies/E-MTAB-5949?query=E-MTAB-5949] (Serra et al., 2018). Raw GBS data for Col x Ler F2 msh2 mutant have been downloaded from ArrayExpress E-MTAB-8252 [https://www.ebi.ac.uk/biostudies/arrayexpress/studies/E-MTAB-8252?query=E-MTAB-8252] (Dluzewska & Blackwell et al., 2020). The Col-0 TAIR10 reference genome is downloaded from the TAIR database [https://www.arabidopsis.org/]. The sequence polymorphism data for the Col/Ler cross used in this study was downloaded from https://1001genomes.org/projects/MPIPZJiao2020/index.html. Seed scoring and pollen scoring raw data generated in this study are provided in the Source data file. Source data are provided with this paper.

Research involving human participants, their data, or biological material

Policy information about studies with human participants or human data. See also policy information about sex, gender (identity/presentation), and sexual orientation and race, ethnicity and racism.

Reporting on sex and gender	N/A
Reporting on race, ethnicity, or other socially relevant groupings	N/A
Population characteristics	N/A
Recruitment	N/a
Ethics oversight	N/A

Note that full information on the approval of the study protocol must also be provided in the manuscript.

Field-specific reporting

Please select the one below that is the best fit for your research. If you are not sure, read the appropriate sections before making your selection.

☒ Life sciences ☐ Behavioural & social sciences ☐ Ecological, evolutionary & environmental sciences

For a reference copy of the document with all sections, see nature.com/documents/nr-reporting-summary-flat.pdf

Life sciences study design

All studies must disclose on these points even when the disclosure is negative.

Sample size	Recombination frequency (RF) was measured in indicated intervals, marked by fluorescent reporter genes using seed-based system (Kbiri et al., 2022, Ziolkowski et al., 2015). The RF is presented in cM and was calculated as a percent of single-color recombinant seeds divided by the total number of seeds derived from F1 plants obtained after crossing fluorescent-tagged lines (FTL) with a non-color accession. For RF and genetic interference measurements using pollen-based system (Yelina et al., 2013), at least 2000 (for CEN3 interval) or at least 8700 (for I3bc double interval) pollen grain events were used for gating and further analyses.<br>Crossover mapping in F2 ColxLer populations was performed based on libraries sent for sequencing: 201 individual libraries for msh2 fancm, 184 for msh2 fancm zip4, 283 for msh2 recq4 and 232 for fancm zip4.
Data exclusions	For RF calculations performed with seed-based system: samples with color/non-color seeds ratio that did not fall within 2.7-3.3 value (based on Mendelian segregation of fluorescent markers), were excluded from analysis.
Replication	RF for each genotype was calculated from at least 5 individuals and from at least 1000 seeds per F1 individual. All attempts at replication were successful.
Randomization	For each experiment, seeds for tested and control individuals were selected randomly, and respective control plants were grown alongside the tested plants. All plants within same experiment were grown in the same growth chamber and with the same growth conditions.
Blinding	For RF measurements, samples were single-blind.

Reporting for specific materials, systems and methods

We require information from authors about some types of materials, experimental systems and methods used in many studies. Here, indicate whether each material, system or method listed is relevant to your study. If you are not sure if a list item applies to your research, read the appropriate section before selecting a response.

Materials & experimental systems

n/a	Involved in the study	
☒	☐	Antibodies
☒	☐	Eukaryotic cell lines
☒	☐	Palaeontology and archaeology
☒	☐	Animals and other organisms
☒	☐	Clinical data
☒	☐	Dual use research of concern
☐	☒	Plants

Methods

n/a	Involved in the study	
☒	☐	ChIP-seq
☐	☒	Flow cytometry
☒	☐	MRI-based neuroimaging

Flow Cytometry

Plots

Confirm that:

- ☐ The axis labels state the marker and fluorochrome used (e.g. CD4-FITC).
- ☐ The axis scales are clearly visible. Include numbers along axes only for bottom left plot of group (a 'group' is an analysis of identical markers).
- ☐ All plots are contour plots with outliers or pseudocolor plots.
- ☒ A numerical value for number of cells or percentage (with statistics) is provided.

Methodology

Sample preparation	Inflorescence from 5-8 individual plants were pooled together and prepared as described in Yelina et al., 2013. Each pool represents one data point on a graph. Briefly, samples were shaken and incubated in appropriate buffers and the resulting solution was filtered to remove any plant debris, leaving only pollen cells.
Instrument	Guava easyCyte 8HT Cytometer (Millipore)
Software	GuavaSoft 3.3 programme (Millipore)
Cell population abundance	Events representing pollen grains were separated based on forward and side scatter and hydrated pollen was gated to exclude dead or damaged material.
Gating strategy	For calculations in CEN3 interval the events were gated into four classes based on their fluorescence emission signals: red, yellow, double-colour, and non-colour. For I3bc interval, which compromises of two adjacent intervals I3b and I3c, events were gated into eight classes, based on their yellow/red/blue fluorescence.

☐ Tick this box to confirm that a figure exemplifying the gating strategy is provided in the Supplementary Information.
